# A Facile Strategy for Preparing Flexible and Porous Hydrogel‐Based Scaffolds from Silk Sericin/Wool Keratin by In Situ Bubble‐Forming for Muscle Tissue Engineering Applications

**DOI:** 10.1002/mabi.202400362

**Published:** 2024-10-20

**Authors:** Elif Beyza Demiray, Tugba Sezgin Arslan, Burak Derkus, Yavuz Emre Arslan

**Affiliations:** ^1^ Regenerative Biomaterials Laboratory, Department of Bioengineering Faculty of Engineering, Çanakkale Onsekiz Mart University Çanakkale 17100 Turkey; ^2^ Stem Cell Research Lab, Department of Chemistry Faculty of Science, Ankara University Ankara 06100 Turkey

**Keywords:** hydrolyzed wool‐keratin, in situ bubble‐forming hydrogel entrapment, muscle tissue engineering, myogenesis, water‐extracted silk sericin

## Abstract

In the present study, it is aimed to fabricate a novel silk sericin (SS)/wool keratin (WK) hydrogel‐based scaffolds using an in situ bubble‐forming strategy containing an *N*‐(3‐dimethylaminopropyl)‐*N*′‐ethylcarbodiimide hydrochloride (EDC) and *N*‐hydroxysuccinimide (NHS) coupling reaction. During the rapid gelation process, CO_2_ bubbles are released by activating the carboxyl groups in sericin with EDC and NHS, entrapped within the gel, creating a porous cross‐linked structure. With this approach, five different hydrogels (S2K1, S4K2, S2K4, S6K3, and S3K6) are constructed to investigate the impact of varying sericin and keratin ratios. Analyses reveal that more sericin in the proteinaceous mixture reinforced the hydrogel network. Additionally, the hydrogels’ pore size distribution, swelling ratio, wettability, and in vitro biodegradation rate, which are crucial for the applications of biomaterials, are evaluated. Moreover, biocompatibility and proangiogenic properties are analyzed using an in‐ovo chorioallantoic membrane assay. The findings suggest that the S4K2 hydrogel exhibited the most promising characteristics, featuring an adequately flexible and highly porous structure. The results obtained by in vitro assessments demonstrate the potential of S4K2 hydrogel in muscle tissue engineering. However, further work is necessary to improve hydrogels with an aligned structure to meet the features that can fully replace muscle tissue for volumetric muscle loss regeneration.

## Graphical abstract

A novel hydrogel‐based bioengineered scaffold with a porous and flexible ultrastructure is fabricated via in situ crosslinking of sericin and keratin. In chorioallantoic membrane analysis, the bioengineered scaffold not only shows angiogenic potential but also promotes the biological behavior of C2C12 muscle cells. These results highlight the potential of the sericin/keratin scaffold for future applications in repairing volumetric muscle tissue loss.

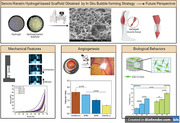

## 1 Introduction

The skeletal muscle, which comprises 30%–40% of the living organism, is the largest dynamic and plastic tissue in the body, providing vital physiological functions such as skeletal movement, posture maintenance, and regulation of body temperature.^[^ ^1^, ^2^ ^]^ Muscle tissue, being a soft tissue, is highly susceptible to damage due to sports trauma and mechanical and biochemical factors, potentially resulting in impaired mobility and internal organ function.^[^ ^1^, ^3^, ^4^ ^]^ Skeletal muscles typically possess a self‐healing capacity in the case of minor injuries by activating resident satellite cells. However, this regenerative capacity proves inadequate in volumetric muscle loss (VML) caused by traffic accidents, war, or tumor ablation, resulting in irreparable loss of muscle function.^[^ ^5^ ^,^ ^6^ ^]^

Nowadays, surgical reconstruction is utilized to treat various muscle tissue damages. Unfortunately, it has shown limited benefits and results due to factors, such as low survival rates, donor‐site morbidity, and shortage of donor sources. Hence, effective strategies for treating muscle injuries are urgently needed.^[^ ^4^ ^]^ Tissue engineering strategies are promising alternatives to the current clinical standard methods to treat damaged or diseased skeletal muscles. Indeed, these strategies aim to create an engineered scaffold that can potentially support and accelerate the healing process.^[^ ^7^ ^]^ The chosen biomaterial should possess fundamental features such as biocompatibility, biodegradability, and the ability to support cellular proliferation and differentiation. Additionally, ensuring suitable micromechanical strength to support contractile functionality is crucial for mimicking native skeletal muscle environments.^[^ ^8^ ^]^ Various biomaterials containing cells and growth factors have recently been developed for skeletal muscle repair. Although some progress has been made, most of these materials cannot tolerate cyclic loading forces consistent with the natural extracellular matrix (ECM).^[^ ^9^ ^]^ Hydrogels, swollen 3D materials, are widely favored as extracellular substrates in muscle tissue engineering due to their tunable mechanical properties, closely resembling native skeletal muscle.^[^ ^10^, ^11^ ^]^ Hydrogels have suitable chemical and mechanical properties and have well‐interconnected micro and/or macropores in their 3D environment to exchange nutrients and metabolites and facilitate cell and tissue ingrowth. In particular, their macroporous structure accelerates angiogenesis by providing enough space for vascular growth, an essential consideration in generating functional tissues.^[^ ^12^ ^]^ Moreover, various techniques, including freeze‐drying, molding, electrospinning, 3D printing, and direct injection into a defect, can be utilized to fabricate well‐designed porous 3D hydrogel‐based scaffolds.^[^ ^11^ ^]^

Hydrogels are typically categorized into natural, synthetic, and hybrid types based on their composition. Natural‐based hydrogels are popular as they often introduce desirable properties to hydrogels, such as biocompatibility, favorable interaction with cells, and inherent bioactivity. However, challenges with using most natural polymers during long‐term cell culture applications include rapid degradation rates and poor mechanical properties.^[^ ^13^ ^]^ To overcome these limitations, modification of various functional groups within the biopolymer repeat units, including amines, carboxyl, and hydroxyl groups, can be applied using several crosslinking methods, such as chemical, light, redox, and thermal. The mechanical properties of formed hydrogels strongly depend on the crosslinked method and degree, as well as biopolymer concentration.^[^ ^14^ ^]^ Hydrogels derived from natural biopolymers like collagen, keratin, gelatin, chitosan, alginate, and silk are extensively studied for skeletal muscle regeneration due to their favorable biochemical properties, flexibility similar to natural tissues, and abundance in nature.^[^ ^15^ ^]^ Keratin hydrogels derived from waste materials, such as hair, nails, feathers, wool, and other epidermal appendages possess several favorable properties, including the promotion of cell migration and differentiation, making them promising candidates for skeletal muscle repair.^[^ ^16^ ^]^ Furthermore, studies have been centered on keratin hydrogels that could facilitate the functional recovery of skeletal muscle in VML injuries through the sustained release of drugs and growth factors. Passipieri et al. developed a human hair keratin‐based hydrogel to serve as a carrier for skeletal muscle progenitor cells and/or growth factors. They evaluated functional recovery in a rat model of VML injury. They also reported significant increases in neo‐muscle tissue formation with the implantation of keratin hydrogel at the defect site.^[^ ^17^ ^]^ Similarly, Baker et al. studied the utility of human hair‐based keratin hydrogels w/o cells and growth factors in treating murine in vivo model VML injury. They stated that promising results in promoting skeletal muscle regeneration.^[^ ^18^ ^]^

Although collagen type I hydrogels can support the natural microenvironment of muscle tissue, their high stiffness limits their effectiveness for long‐term muscle culture, differentiation, and contractile force generation, which are key challenges in vitro skeletal muscle tissue engineering applications.^[^ ^19^ ^]^ Other limitations of its use in this field include rapid degradation,^[^ ^20^ ^]^ cost,^[^ ^21^ ^]^ batch variability,^[^ ^22^ ^]^ and immune responses.^[^ ^23^ ^]^ In contrast, silk‐based hydrogels have the potential for musculoskeletal system regeneration. Their surface nanopatterning and tunable mechanical properties, obtained by chemical modifications through reactive amino acids in the silk fibroin,^[^ ^24^ ^]^ make them ideal for this purpose. However, the main challenging issues are high brittleness,^[^ ^25^ ^]^ processing complexity, and rapid degradation,^[^ ^26^ ^]^ limiting its usage in muscle tissue engineering applications. On the other hand, silk sericin (SS) emerges as an important alternative compared to other natural polymers when evaluated from this perspective. SS is a natural polymer produced by the silkworm Bombyx mori, constituting 25%–30% of the total cocoon weight. It surrounds and holds fibroin fibers together owing to its gummy structure for constructing cocoon shells.^[^ ^27^ ^]^ Additionally, SS, which contains high amounts of polar amino acids, such as serine, aspartic acid, and threonine in its molecular structure, can easily form adhesive and gel‐forming properties through hydrogen bonding with the surrounding water molecules.^[^ ^28^ ^]^ It has been utilized in numerous biomedical applications in different forms, such as hydrogels, films, sponges, and scaffolds, for its exceptional biological activities, low immunogenicity, and suitable cell‐adhesion interaction.^[^ ^29^ ^]^ Research has indicated that sericin, due to its natural composition and structure, shows promising effects in drug delivery, wound dressing, and bone grafting.^[^ ^28^ ^]^ On the other hand, to our knowledge, only one study related to SS in the field of muscle tissue engineering was published by Song et al. They designed a sericin patch delivering miR29‐genetically modified myoblasts for rapid regeneration and functional repair of severe skeletal muscle injury. Based on their findings, the sericin patch enhanced the survival and proliferation rate of the transplanted myoblasts. Moreover, the sericin patch exhibited an optimum degradation rate and highly porous structure to allow myoblast fusion.^[^ ^30^ ^]^ Here, we aimed to produce novel hydrogel‐based scaffolds from SS/WK using an in situ bubble‐forming hydrogel entrapment process. These materials are designed to have all‐in‐one properties crucial for mimicking the native muscle tissue environment, including biochemical cues, porosity, and suitable mechanical strength to support contractile function. This methodology obtained cross‐linked hydrogels via a well‐known EDC/NHS coupling reaction where an amide bond formed between the ‐carboxyl and ‐amine functional groups in the proteinaceous mixture. During the in situ gel formation, CO_2_ bubbles formed as a result of activating the carboxyl groups in the SS with EDC/NHS, which were then entrapped in the gel, allowing a porous structure within the material. Hydrogels containing SS and WK with different ratios were prepared to study them comparatively based on their mechanical and rheological behaviors and compression‐recovery process. In ovo chick chorioallantoic membrane (CAM) assay was conducted to assess the biocompatibility and proangiogenic response of the hydrogels. The effects of developed hydrogels on myoblast survival and myogenic differentiation capacity were tested by Live‐dead and MTS assays, histochemical (HC) staining, and gene expression levels.

## 2 Results and Discussion

### 2.1 Biochemical Evaluations of SS and WK Extracts

The concentration of the regenerated SS solution was calculated to be 6.067% ± 0.006% w/v using the Lowry standard calibration curve given in our previous study.^[^ ^31^ ^]^ Extraction techniques, such as boiling with water, degumming in sodium carbonate (Na_2_CO_3_) solution, acidic and alkali media, boiling‐off in soap, and enzymatic treatment are extensively used to recover sericin.^[^ ^32^ ^]^ In this study, sericin was extracted from silk cocoons using a boiling method that does not require any chemical agents, complicated equipment, or purification process. Moreover, extraction method, time, and temperature significantly affect the biochemical properties, such as peptide chain size, amino acid content, and structural conformation, as well as the physicochemical and biological activity of the sericin.^[^ ^33^ ^]^

Keratin content in the dialysate was calculated as 12.19±0.11 mg mL^−1^ by using the calibration curve given in Figure S1‐A (Supporting Information). Additionally, freeze‐dried WK extract yielded ≈42.5% ± 0.3%. The sulfide bridges formed between cysteine residues in peptides were broken via a sulfitolysis reaction to give cysteine thiol (cysteine‐SH) and S‐sulfonated residues. These residues were then analyzed using Ellman's reagent quantitating free sulfhydryl groups and found as 0.20 ± 0.07 mmol mg^−1^ keratin using Equation (1) obtained from the L‐cysteine calibration curve (Figure S1‐B, Supporting Information). All findings regarding the biochemical properties of keratin are in close agreement with our previous studies.^[^ ^31^, ^34^ ^]^

Sodium dodecyl sulfate‐polyacrylamide gel electrophoresis (SDS‐PAGE) analysis was conducted to determine the Mw distributions of peptide chains in SS and WK extracts (Figure S1‐C, Supporting Information). To investigate the effect of different extraction methods on protein integrity and to evaluate the results comparatively, Na_2_CO_3_‐extracted SS, which is obtained by boiling aqueous 0.02 m Na_2_CO_3_ solution for 15 min, was also included in the electrophoretic profile. The SDS‐PAGE pattern of water‐extracted SS showed a large smear band at around 200 kDa and a distinct band at around 20 kDa. On the other hand, a smeared band between 6 and 20 kDa was observed in the Na_2_CO_3_‐extracted SS. These results have indicated that the sericin can be hydrolyzed in aqueous media in small fractions by cleaving peptide bonds. The basic solution promotes protein hydrolysis, which explains why the Na_2_CO_3_‐extracted SS has a smaller Mw.^[^ ^35^ ^]^ Moreover, the water solubility of both freeze‐dried extracts was investigated, revealing that Na_2_CO_3_‐extracted SS was dissolved easily in cold water, while water‐extracted SS was only dissolved in hot water at about 65 °C. In addition, many attempts have been made to obtain cross‐linked gels using Na_2_CO_3_‐extracted SS with small fractions, but hydrogel formation was not achieved. This solubility profile and reactivity with other substances are related to their peptide chain size and determine the usage area of sericin. Sericin with lower Mw (≤20 kDa) can be used in biomaterials, health, and cosmetics productions, such as skincare and haircare products; on the other hand, sericin with high‐Mw (≥20 kDa) can be used in tissue engineering applications in different forms including biomaterials, polymers, functional bio‐membranes, and hydrogels.^[^ ^33^, ^36^ ^]^ Based on the mentioned results and features, water‐extracted SS with high Mw of peptides was used for hydrogel formation.

The SDS‐PAGE pattern of the WK extract shows small peptide fractions with an Mw of about 6–8 kDa, revealing that the highly alkaline media (pH 12−14) during extraction damages the structural integrity of keratin. In our previous study, the Mw distribution of the peptide chains in the keratin was more accurately determined as 2–3 kDa using matrix‐assisted laser desorption/ionization‐time of flight (MALDI‐TOF) analysis. It was also reported that many different‐sized keratin fractions are essential for triggering hydrogel formation due to the presence of functional groups.^[^ ^31^ ^]^

### 2.2 Constructed Scaffolds and their Physicochemical Evaluations

SS/WK hydrogels were prepared using the in situ bubble‐forming hydrogel entrapment strategy, employing EDC/NHS reagents as cross‐linkers to facilitate amide bonds between carboxyl and amine groups within the proteinaceous structure. During the process, an amide reaction between sericin and keratin occurred catalyzed by EDC/NHS. The carboxyl groups in sericin were activated by EDC/NHS, leading to the rapid generation of unstable intermediates and CO_2_ bubbles. The EDC/NHS coupling reaction is widely utilized in the literature and involves the formation of amide bonds between carboxylic and amine groups in peptides and proteins. In this mechanism, EDC activates the carboxyl groups in the polymer chains to form O‐acylisourea. This unstable intermediate can subsequently react with free amino groups to form amide bonds, undergo hydrolysis, or rearrange into O‐acylisourea residues. The addition of NHS enhances reaction efficiency by stabilizing the O‐acylisourea intermediate and converting it into a semistable amine‐reactive NHS ester.^[^ ^37^ ^]^ According to our observations during the experiment, as the amount of sericin protein increases, the gas bubble that comes out also increases at that scale. This is because sericin contains a high amount of aspartic acid, which includes an additional carboxyl group in its structure. When evaluated in this context, when an increasing amount of EDC is added to the structure as the protein ratio, the carboxyl groups are activated, and CO_2_ bubbles are released, which explains the increasing amount of CO_2_ bubbles with the amount of sericin. A study indicated a similar result. The authors claimed that EDC activated carboxyl groups of hyaluronic acid, which was responsible for CO_2_ bubbles.^[^ ^12^ ^]^

On the other hand, in the current literature, there are other explanations for this phenomenon. In fact, the EDC/NHS coupling reaction by itself does not produce any gaseous byproducts; CO_2_ bubbles could form due to the reaction of trace amounts of ethyl isocyanate (one of the educts of EDC synthesis) in a manner. In this context, using EDC at high concentrations may have led to the formation of more gas bubbles.^[^ ^38^ ^]^ In addition, in the described method, gas bubbles began to form immediately upon adding EDC to the viscous proteinous mixture containing NHS, indicating that EDC could be responsible for CO_2_ formation.

The sol–gel transformation was completed for 5–10 min, and meanwhile, the CO_2_ bubbles were entrapped in the viscous gel, resulting in a porous network formed inside the material. Hydrogels were left to rest at RT for 3 h for maturation. In this experimental setup, WK and SS were tested alone or combined in different ratios and weights (S1K2, S2K1, S4K2, S2K4, S6K3, S3K6) to determine the hydrogel with the most suitable structural, mechanical, and rheological properties. Among them, hydrogels could not be obtained using individual WK and SS with different weights, while the S1K2 hydrogel with a too fragile structure to be handled was obtained (data not shown). The fact that the reactions depended on the presence of both protein extracts suggested that a cross‐linked hydrogel network structure was formed by enclosing the small‐Mw keratin fractions by the higher‐Mw sericin peptide chains, most of which have strongly polar side groups like hydroxyl and carboxyl. Simply put, keratin serves as a bridge to bring together the large chains in the sericin. On the other hand, using both alone is insufficient to facilitate coupling reactions between peptide fragments in their structure.

#### 2.2.1 Rheological Analysis

**Figure 1:**
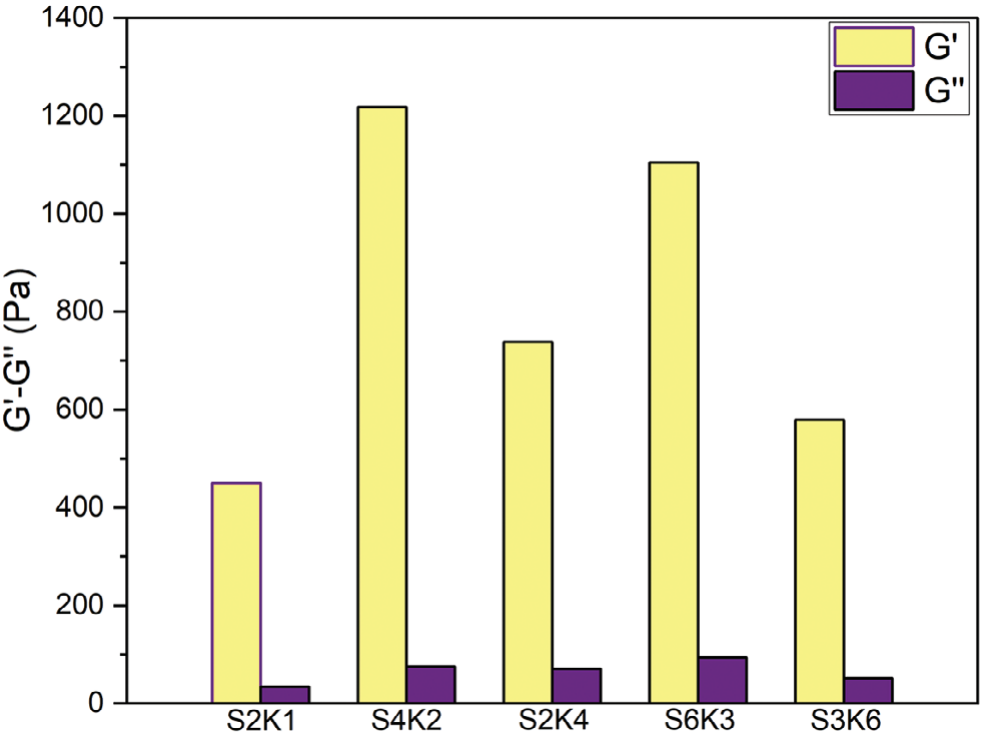
Storage (*G*′) and loss (*G*″) modulus values extracted from frequency sweep analyses (1 Hz) performed for hydrogels having different sericin/keratin ratios (S2K1, S4K2, S2K4, S6K3, and S3K6).

Dynamic oscillatory rheology was used to evaluate the viscoelastic behavior of the sericin/keratin hydrogels, including different ratios and weights of SS and WK extracts. Initially, linear viscoelastic Region (LVR) was determined at a strain value of 4% from the amplitude sweep at 1 Hz. During the dynamic frequency, temperature, and time sweep tests, storage modulus (*G*′) and loss modulus (*G*″) were measured to assess the gel solid‐like (elastic) and the liquid‐like (viscous) behavior of the samples, respectively. Furthermore, a gel with a higher *G*′ value generally exhibits better mechanical and structural properties.^[^ ^39^ ^]^ **Figure** **1** shows the frequency dependence of dynamic moduli (*G*′ and *G*″) for all matured hydrogels. *G*′ was greater than *G*″ throughout the frequency range tested, indicating the gel‐like behavior of all samples. It was clearly seen from Figure S3‐A (Supporting Information) that an increase in sericin and total protein content in the hydrogels resulted in higher *G*′ values. Additionally, the *G*′ values for S4K2 and S6K3 were 1200 and 1100 Pa, respectively, suggesting S4K2 hydrogels are slightly stiffer than S6K3 hydrogels. These observations indicate that after reaching a certain threshold, further increases in the total protein amount do not significantly contribute to the gel's stiffness.

An oscillatory temperature sweep was conducted at 25–40 °C with a heat rate of 1 °C min^−1^ to determine the mechanical properties of matured hydrogels as a function of temperature (Figure S3‐B, Supporting Information). No significant changes in *G*' and *G*“” values were observed with increasing temperature, reflecting that all samples maintained their 3D gel or solid‐like structures, especially under body‐temperature conditions.

Rheological analysis was also used to monitor the gelation process of S2K4 and S4K2 hydrogels for 30 min. For this purpose, *G*' and *G*“” values as a function of time were measured by subjecting pregel forms of samples to an oscillatory time sweep at 25 °C, 1 Hz frequency, and 7.5% strain. Due to the rapid nature of the reaction, the gelation point, indicated by the intersection of the two moduli, could not be precisely determined. The crossover point (*G*′ = *G*″) divides the gelation process into two regions: predominantly viscous (*G*′ < *G*″) and predominantly elastic (*G*′ > *G*″).^[^ ^40^ ^]^ However, we could not identify this cross‐over point since *G*′ predominated over *G*″ at the start of the gelation process and these moduli for both hydrogels rapidly increased as the gelation progressed (Figure S3‐C, Supporting Information). The sharp increase in both moduli observed in the S2K4 and S4K2 hydrogels reached a plateau at the 10th and 5th min of the crosslinking reaction, indicating that the reaction kinetics accelerated with increasing amounts of sericin in the hydrogels. At the 25th minute of the final network formation, S4K2, with a higher *G*' value (1000 Pa) than S2K4 (100 Pa), exhibited better mechanical strength.

In addition, compared to S2K4, the difference between the elastic and viscous modulus in S4K2 during gelation is significantly greater, indicating that it has a more gel‐solid behavior. In a study, Vulpe et al. investigated the influence of added sericin to hyaluronic acid (HA) and/or collagen on gelation via in situ crosslinking reaction. The authors showed that reaction kinetics became faster in the presence of sericin, which offers possibilities of amide bond formation.^[^ ^41^ ^]^ These results are good confirmation that the higher amount of sericin in the structure significantly contributed to gelation by accelerating amide bond formation.

#### 2.2.2 Micromechanical Evaluation

**Figure 2:**
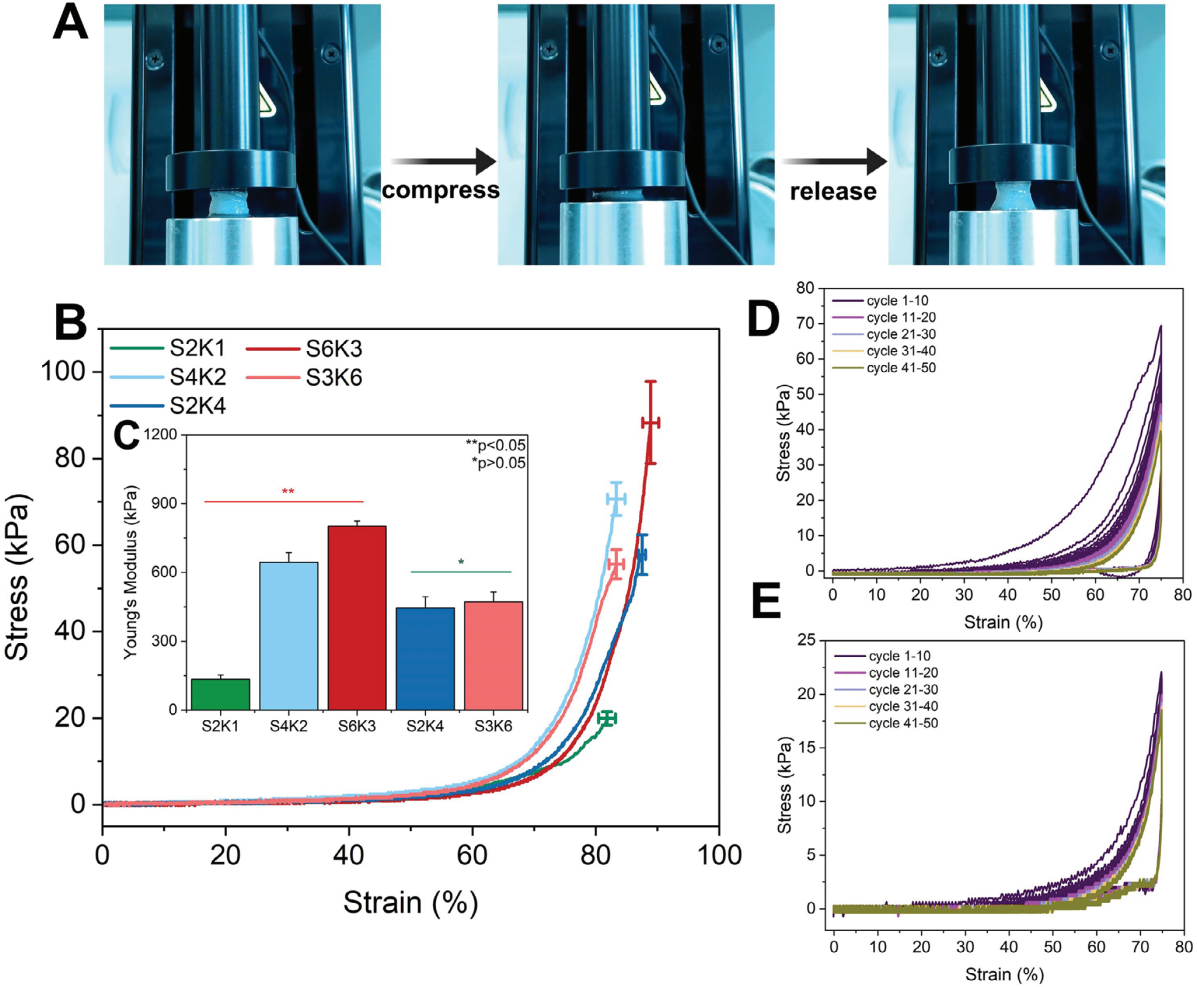
Micromechanical analysis results. Compressive testing images A), The stress–strain curves B), and Young's modulus values C) for five different sericin/keratin hydrogels. Cyclic diagrams related to 50 consecutive cyclic loading–unloading experiments for S4K2 D) and S2K4 E) hydrogels.

The mechanical properties of the hydrogels were examined through uniaxial compression tests (**Figure** **2**). Table S1 (Supporting Information) summarizes compressive stress, strain (%), and Young's modulus values. As both the sericin ratio and the total protein mass in sericin/keratin hydrogels increased, compressive stresses and Young's modulus values also increased. The compressive stress increases observed between S2K4 (57.1±0.71 kPa) and S4K2 (69.33 ± 1.35 kPa) hydrogels and S3K6 (52.45 ± 3.45 kPa) and S6K3 (91.63 ± 9.46 kPa) hydrogels are attributed to the increasing sericin content. On the other hand, the increase in total protein mass can explain why S6K3 is more robust than S4K2 despite having the same sericin ratio. Moreover, the compressive stress values of S2K4 and S3K6 hydrogels with the same sericin ratios are not significantly different (*p* < 0.05), indicating that protein increase alone is not a determining factor of mechanical strength. Furthermore, S2K1 hydrogels showed significantly lower compressive strength at 19.24 ± 1.52 than others due to inadequate total protein mass. All of the sericin/keratin hydrogels exhibited comparable strains within the 81%–89% range, suggesting that they have good compression‐bearing capacity. As mentioned, sericin supports building a hydrogel network by accelerating amide bond formation during the crosslinking reaction. Mechanical strength depends on the amount of protein and the crosslinking density.^[^ ^42^ ^]^ Similar mechanical test results were found in the studies that obtained hydrogels by adding Alginate^[^ ^43^ ^]^ and poly(ethylene glycol) diacrylate (PEGDA)^[^ ^42^ ^]^ to silk sericin. They attributed the increase in mechanical reinforcement to the increased ratio of sericin in the hydrogel.^[^ ^42^, ^43^ ^]^ In another study, Wang et al. showed that sericin hydrogels have high compressive strength and stated that they can be used to regenerate tissues like myocardium and skeletal muscle.^[^ ^44^ ^]^ It is important to note that materials should have similar intrinsic mechanical properties as native muscle to support normal muscle function in the anatomical site of implantation and should be sufficiently robust to be handled during the surgical process. In this context, Young's modulus values (or elastic modulus) of our hydrogels, which range from 134 to 471 kPa, are considerably higher than those of native skeletal muscle tissues (20–100 kPa).^[^ ^45^ ^]^ These results suggest that all the hydrogels can withstand dynamic mechanical loads in vivo. However, as stated before, the elastic modulus of the hydrogels can be tuned to be similar to that of native skeletal muscle by changing the amount of sericin in the hydrogel networks and the degree of crosslinking.

During compressive stress–strain analysis, we observed that water‐saturated hydrogels released the water they contained under compression and quickly returned to an initial shape by absorbing the water around them after the removal of compression (Figure 2A; and Video S1, Supporting Information). This observation indicates that hydrogels with porous structures have elastic forces owing to their ability to swell and release water. Among all the hydrogels, S2K4 and S4K2 hydrogels could recover the shapes after the compression test. Therefore, cyclic loading–unloading experiments were conducted to examine the further mechanical behavior of both hydrogels by subjecting the hydrogels to 50 consecutive cycle tests, and the cyclic diagrams were obtained (Figure 2D,E). When the area between the loading–unloading curves increases, the dissipated energy per unit volume increases, resulting in a higher level of hysteresis. Conversely, a decrease in this area leads to reduced dissipated energy and hysteresis levels, suggesting a higher material recovery rate after unloading.^[^ ^46^ ^]^ In the first cycle, the S4K2 hydrogel showed a noticeable hysteresis loop due to the sudden removal of CO_2_ bubbles within the hydrogel under compression, resulting in high dissipated energy (Figure 2D). However, almost zero hysteresis was observed within 40 cycles, indicating no obvious dissipated energy and successful recovery of the hydrogel to its original shape by maintaining its integrity. An increase in hysteresis was observed in the 41–50 cycles, indicating permanent deformation of the hydrogel and a lower recovery rate. S2K4 hydrogel also exhibited similar recovery properties in the same loading and unloading cycles with lower stress values (Figure 2E). Additionally, S2K4 had a lower hysteresis loop in the first cycle than S4K2. It can be suggested that fewer CO_2_ bubbles formed inside the S2K4 hydrogel with less sericin content led to lower dissipated energy observed during the compression test. Given its elasticity and suitable mechanical properties, the S4K2 hydrogel was selected for this study's follow‐up experiments and analysis. Additionally, S2K4 hydrogel was used in subsequent experiments to demonstrate the effect of sericin and compare results.

#### 2.2.3 ATR‐FTIR Results

**Figure 3:**
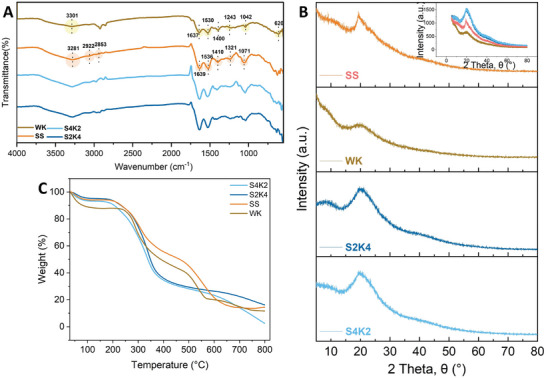
ATR‐FTIR spectra A), XRD patterns B), and TGA curves C) of WK, water‐extracted SS, S2K4, and S4K2 hydrogel‐based scaffolds.

ATR‐FTIR spectroscopy was utilized to detect functional groups based on the vibration of repeating units in the protein structures. **Figure** **3**A illustrates characteristic absorption bands of the keratin, SS, and hydrogel‐based scaffolds. Amid A bands observed between 3200 and 3400 cm^−1^ attributed to H‐bonded N−H stretching vibration. Amid I‐III bands interpreted in terms of conformational changes in structures of proteins and polypeptides are also seen in all spectra. The amide I band at 1600–1700 cm^−1^ and amide II bands at 1500–1600 cm^−1^ correspond to the C═O stretching and N*─*H deformation with some contributions of C*─*N stretching, respectively. On the other hand, peaks at 1200−1300 cm^−1^ that show amide III bands are related to C−N and C−C stretching absorptions and N−H and C−O bending absorptions.^[^ ^31^, ^34^ ^]^ Among them, the amide I band is particularly useful in elucidating the secondary structure of sericin.^[^ ^43^ ^]^ The observation of a stronger amide I peak in sericin compared to keratin may be attributed to differences in secondary structures. Furthermore, the intensity of the amide I peak in the S2K4 and S4K2 hydrogels slightly increased, suggesting structural transformations occurred in the sericin/keratin‐based hydrogel network after the reaction.

#### 2.2.4 XRD Analysis

To better understand structural changes after the reaction, XRD analysis was also performed. Figure 3B shows the XRD pattern of all the samples. SS exhibited a strong crystal surface diffraction peak around 2θ = 19.2°, relating to the β‐sheet structure resulting from intermolecular hydrogen bonds formed between the hydroxyl groups in the amino acid residues of SS extracts obtained after the degumming process.^[^ ^47^ ^]^ An amorphous diffraction peak was seen at ≈2θ = 20.0° in the spectrum of WK, indicating β‐sheet structures. On the other hand, the characteristic peak around 2θ = 9° corresponding to the α‐helix structures of natural WK was not seen due to damage to these structures in WK extracts by an alkaline treatment during the sulfitolysis reaction.^[^ ^34^, ^48^ ^]^ S2K4 and S4K2 hydrogels had broad and amorphous peaks around 2θ = 19.8°, with higher intensity than pure WK and SS, indicating the success of hydrogel preparation. During the formation of the hydrogel network, small amorphous WK fractions were incorporated between the polypeptide chains of the sericin in the crystal structure, resulting in the whole hydrogel being an amorphous structure.

#### 2.2.5 Thermal Stability

Thermal degradation of all samples was investigated using TGA (Figure 3C). Thermogravimetric curves of the S2K4 and S4K2 hydrogels show two decomposition stages, whereas the pure SS and WK extracts show three. The initial stage of weight loss below 100 °C is attributed to the loss of bound water. The second and third stages of weight loss in the temperature range of 300–600 °C are mainly due to the degradation of proteins and further pyrolysis of the degraded products, respectively.^[^ ^49^, ^50^ ^]^ At 500 °C, the weight losses for WK and SS are 60% and 50%. Additionally, S2K4 and S4K2 hydrogels have similar thermograms, with weight loss of 70% at the same temperature. Composite hydrogels exhibited reduced thermal stability compared to the pure protein extracts. This result is closely related to the degree of loss of crystallinity, as corroborated by XRD analysis. An amorphous network hydrogel was obtained due to the incorporation of small amorphous WK fractions between the sericin polypeptides during the crosslinking reaction, which disordered the crystalline structure of the SS. A decrease in crystal‐sheet structures, where intermolecular interactions between protein chains are stronger, makes the hydrogel sensitive to thermal processes and reduces thermal stability.^[^ ^50^, ^51^ ^]^

#### 2.2.6 Morphology, Porosity, Swelling, Wettability Properties, and EDX Analysis

SEM analysis was used to investigate the pore structure and pore size distribution of the hydrogel‐based scaffolds (Figure S4‐A, Supporting Information). All micrographs clearly show interconnected porous structures and high porosity within the materials, revealing the successful preparation of hydrogel networks via an in situ bubble‐forming gelation process. In other words, pore formation inside the hydrogel‐based scaffolds was achieved by removing entrapped CO_2_ bubbles formed during the EDC/NHS coupling reaction and water content in the gels by lyophilization technique. In addition, the mean pore sizes of the S2K4 and S4K2 scaffolds calculated from SEM images were found to be 66.33 ± 1.78 and 93.87 ± 1.63 µm, respectively. In the EDC spectra presented in Figure S4‐B (Supporting Information), carbon, oxygen, nitrogen, and sulfur were detected on both hydrogel‐based scaffolds, confirming the presence of SS and WK in the structure. Moreover, the sulfur content is attributed to a high concentration of sulfur‐containing amino acids, such as methionine and cysteine, which are abundant in the water‐extracted SS and WK.^[^ ^33^, ^34^ ^]^

Moreover, the total surface areas of the S2K4 and S4K2 hydrogel‐based scaffolds calculated by the BET method were found to be 6.66 and 43.61 m^2^ g^−1^, respectively (Table S2). These results indicated that hydrogels with larger pore sizes and increased total surface areas were obtained by adding more sericin. For the design of materials intended for skeletal muscle regeneration, it is essential to have an optimal pore size that allows for cell nutrition, proliferation, and migration throughout the entire scaffold. Furthermore, the material's porosity and structure should be appropriate to enhance the fusion and differentiation of myoblasts into myotubes, which are crucial for effective muscle regeneration.^[^ ^52^, ^53^ ^]^ Many studies have investigated the effect of pore size on myoblast behaviors, such as proliferation and differentiation. In a study reported by Kozan and co‐workers, collagen sponges with pore sizes in the range of 76–89 µm were produced by their custom freezing method applied at temperatures, such as −20, −40, −60, −80, and −196 °C. They also revealed that the collagen scaffolds produced at −20 °C with a diameter of 88.9 ±17.6 µm showed the best supportive effect on myoblast infiltration and differentiation.^[^ ^52^ ^]^ In another study, cryogels containing gelatin‐carboxymethyl cellulose materials with pores ranging from 30 to 75 µm in diameter were produced via a cryogelation process for skeletal muscle regeneration. Researchers stated that mature myotubes typically have a width not exceeding 30 µm and also reported that their cryogels with alignment structures had optimum pore sizes to facilitate myoblast infiltration and differentiation into skeletal muscle myotubes.^[^ ^53^ ^]^ Based on these findings, we concluded that our S4K2 hydrogel‐based scaffold can provide an optimal pore size and structure for favorable myoblast behavior.

The materials' swelling properties are another vital factor in the absorption of nutrients and other fluids from the extracellular microenvironment, consequently affecting the cell survival rate. These properties depend on several features of the materials, such as porosity, hydrophilic character, and crosslinking ratio.^[^ ^31^, ^54^ ^]^ Therefore, the swelling ratios of the S2K4 and S4K2 hydrogel‐based scaffolds were determined by the PBS uptake test and found to be 1400.17% ± 107. %and 1615.06%±38.52%, respectively (Figure S5‐A, Supporting Information). As the SS content increased, the S4K2 hydrogel's porosity, surface area, and hydrophilic nature increased, resulting in a higher swelling ratio compared to the S2K4 hydrogel.

Contact angle measurements were carried out to determine the water retention capacity of the material surface through further analysis. During the wettability test of the S4K2 hydrogel‐based scaffold surface, a small drop of water on the surface was immediately absorbed, indicating its highly hydrophilic nature (Video S2, Supporting Information). The swelling and porosity properties also corroborated these results.

### 2.3 In Vitro Assessment of the Enzymatic Degradation

A suitable scaffold should meet the correct balance of functional properties and biodegradation rate to ensure proper remodeling of the damaged tissue after implantation. Biodegradable scaffolds must maintain their structural integrity until holding cells in place and degrade in a timely manner that matches that of new tissue growth.^[^ ^55^ ^]^ Therefore, degradation rates of both hydrogel‐based scaffolds were calculated as a percentage of weight loss by following a protease treatment for 3, 7, and 10 days of incubation (Figure S5‐B, Supporting Information). At the end of each time point, the S4K2 hydrogel showed a slightly higher degradation rate than the S2K4 hydrogel. On the 10th day, the total mass loss of S2K4 and S4K2 hydrogels reached 91.72% ± 4.67% and 96.63% ± 2.43%, respectively. The biodegradation process is a complex phenomenon that depends on several factors, such as pore size, surface area, hydrophilicity, porosity, and swelling ratio.^[^ ^56^ ^]^ A slight increase in the degradation rate of the S4K2 hydrogel can be attributed to the mentioned material features. S4K2 hydrogel exhibited a higher liquid uptake capacity due to its superior pore structure and swelling ratio, rendering it more susceptible to hydrolytic attacks, leading to an increased degradation rate.^[^ ^31^ ^]^

### 2.4 In Ovo CAM Assay

**Figure 4:**
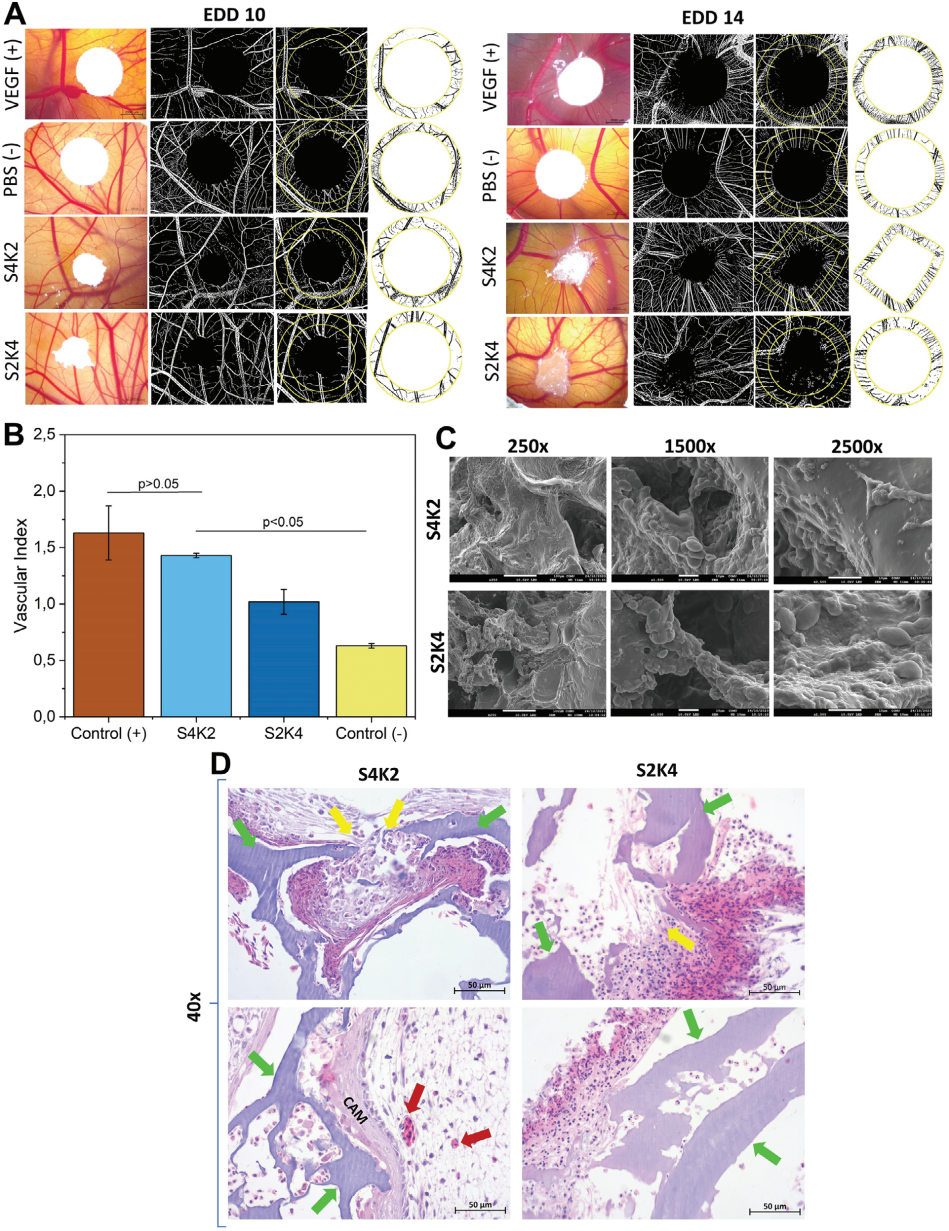
In ovo CAM assay for assessment biocompatibility and proangiogenesis of S4K2 and S2K4 hydrogel‐based scaffolds. Stereomicroscopic and binary images of the implanted material on EDD10 and EDD14 A). Vascular index graph B), SEM micrographs (white scale bars represent 100 µm for 250x and 10 µm for 1500x and 2500x) C), and histological images D) of tissue‐CAM complex sections at EDD14. In the histological images, green arrows indicate CAM‐hydrogel complex, while red and yellow arrows indicate newly formed blood vessels and cell migration, respectively. Scale bars represent 50 µm (40X).

The in ovo CAM assay is a valuable model in tissue engineering applications for studying the angiogenic response, which involves the formation of new blood vessels delivering nutrients and oxygen to neighboring cells, as well as the biocompatibility of materials.^[^ ^57^ ^]^ Therefore, the proangiogenic properties and biocompatibility of S2K4 and S4K2 hydrogel‐based scaffolds were evaluated using the CAM assay in vivo animal model through microscopic and histological observations (**Figure** **4**A–D). Stereomicroscopic images revealed that both hydrogels retained their structural integrity after 4 days of incubation in the CAM (Figure 4A). The vascular density and indexes were calculated from the depicted area in the binary images with a black‐white scale obtained by the Image J software (Figure 4B). The statistical analysis showed no significant differences between the vascular index values of the S4K2 hydrogel‐based scaffold and VEGF (control +). On the other hand, the index of the S4K2 hydrogel‐based scaffold was found to be higher than those of S2K4 and PBS (control ‐) by 1.4 and 2.27 folds, respectively. We reasoned that S4K2 hydrogel enhanced cellular infiltration and new blood vessel formation related to its higher surface area and porosity.^[^ ^34^ ^]^

SEM micrographs shown in Figure 4C revealed cellular migration, attachment, and the existence of microvessels on both hydrogel‐CAM tissue sections.

H&E‐stained hydrogel‐CAM tissue sections demonstrated the cellular and vascular structures, revealing that both hydrogels provide a supportive environment for cell infiltration and proliferation. The regions indicated by yellow arrows provide evidence for cell migration into the CAM‐hydrogel tissue constructs indicated by green arrows. Blood cells and newly formed vessels (represented by red arrows) grooving toward the CAM‐hydrogel complex were also observed (Figure 4D). These results suggest that hydrogels exhibit good integration by surrounding tissues and pro‐angiogenic properties. Additionally, we confirmed that both hydrogel‐based scaffolds did not cause any adverse effects, such as inflammatory reactions, necrosis, or other unfavorable tissue responses, indicating their biocompatibility.

### 2.5 Cell Culture Studies and Histochemical Assessments

**Figure 5:**
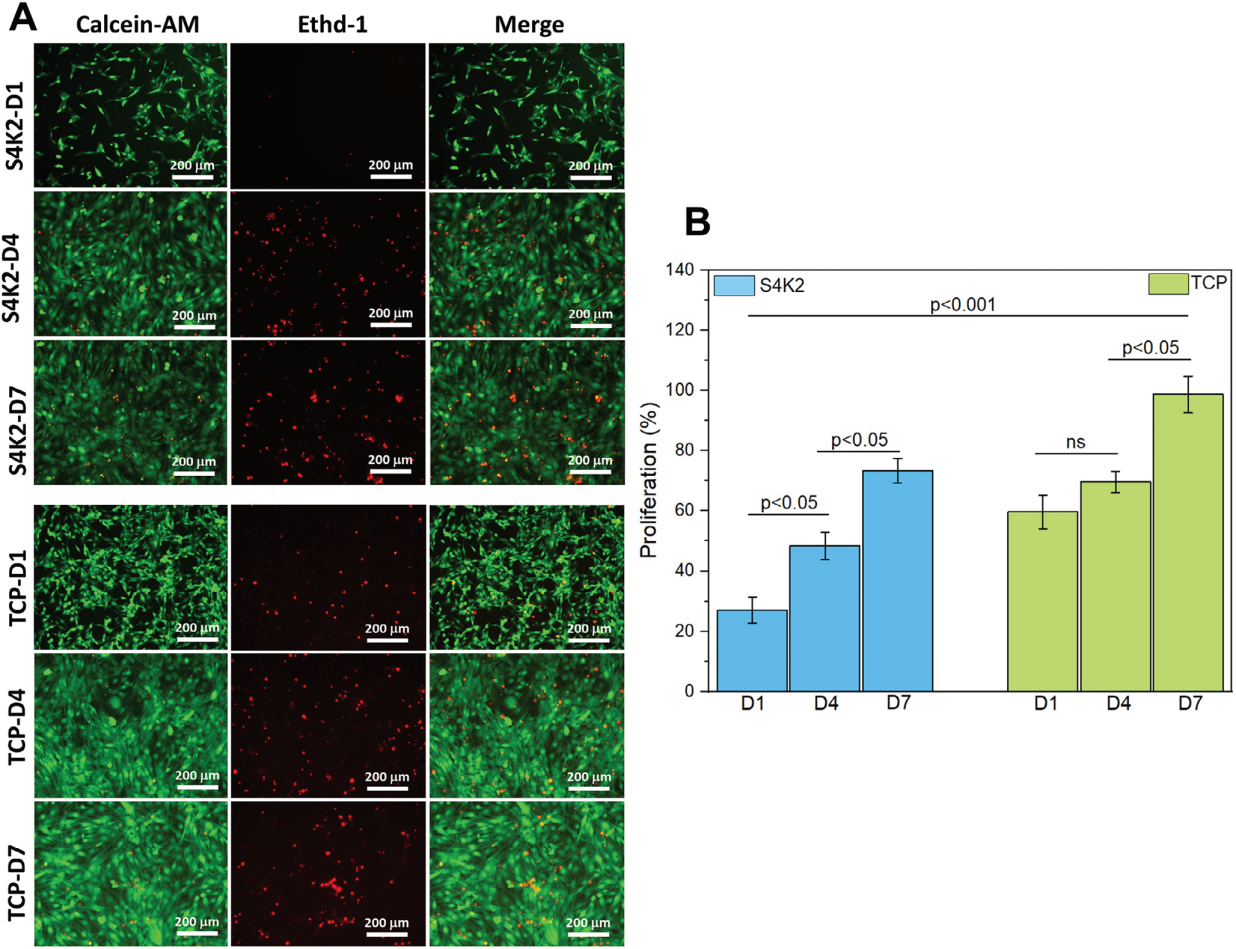
Cytocompatibility test for the S4K2 hydrogel‐based scaffold. In the fluorescent microscopic images, green staining represents live cells, while red staining represents dead cells. The white scale bar is 200 µm A). MTS assay results regarding C2C12 cell proliferation on S4K2 hydrogel‐based scaffolds for 1, 4, and 7 days (ns = not significant) B).

Live/dead assay was performed to determine the cytocompatibility of S4K2 hydrogel‐based scaffolds. Calcein‐AM/EthD‐1 double staining fluorescent images of C2C12 myoblasts after 1, 4, and 7 days of culture were shown in **Figure** **5**A. A comparison was made with an accepted standard in vitro culture material of TCP to asses cell adhesive properties. Representative microscopy images showed (green emission) an increased level of living cells on the S4K2 hydrogel‐based scaffold throughout the all‐time points, suggesting that they are cell‐friendly. On the other hand, green fluorescent emissions were stronger for attached cells on TCP (positive control) compared to the scaffold due to the infiltrated cells into the porous S2K4 hydrogel‐based scaffold which could not be visualized.

To better assess the cell compatibility of the S4K2 hydrogel‐based scaffolds, MTS test was also performed. Results, as shown in Figure 5B, were consistent with the live/dead assay and indicated that the S4K2 hydrogel‐based scaffolds with porous structure provided a biocompatible microenvironment for C2C12 cells. Furthermore, the increased cell density over a 7‐day proliferation period on the hydrogel‐based scaffold was lower than that on TCP, suggesting a potential differentiation of myoblasts rather than proliferation,^[^ ^52^ ^]^ which was also confirmed by quantitative RT‐PCR analysis as explained in next sections.

**Figure 6:**
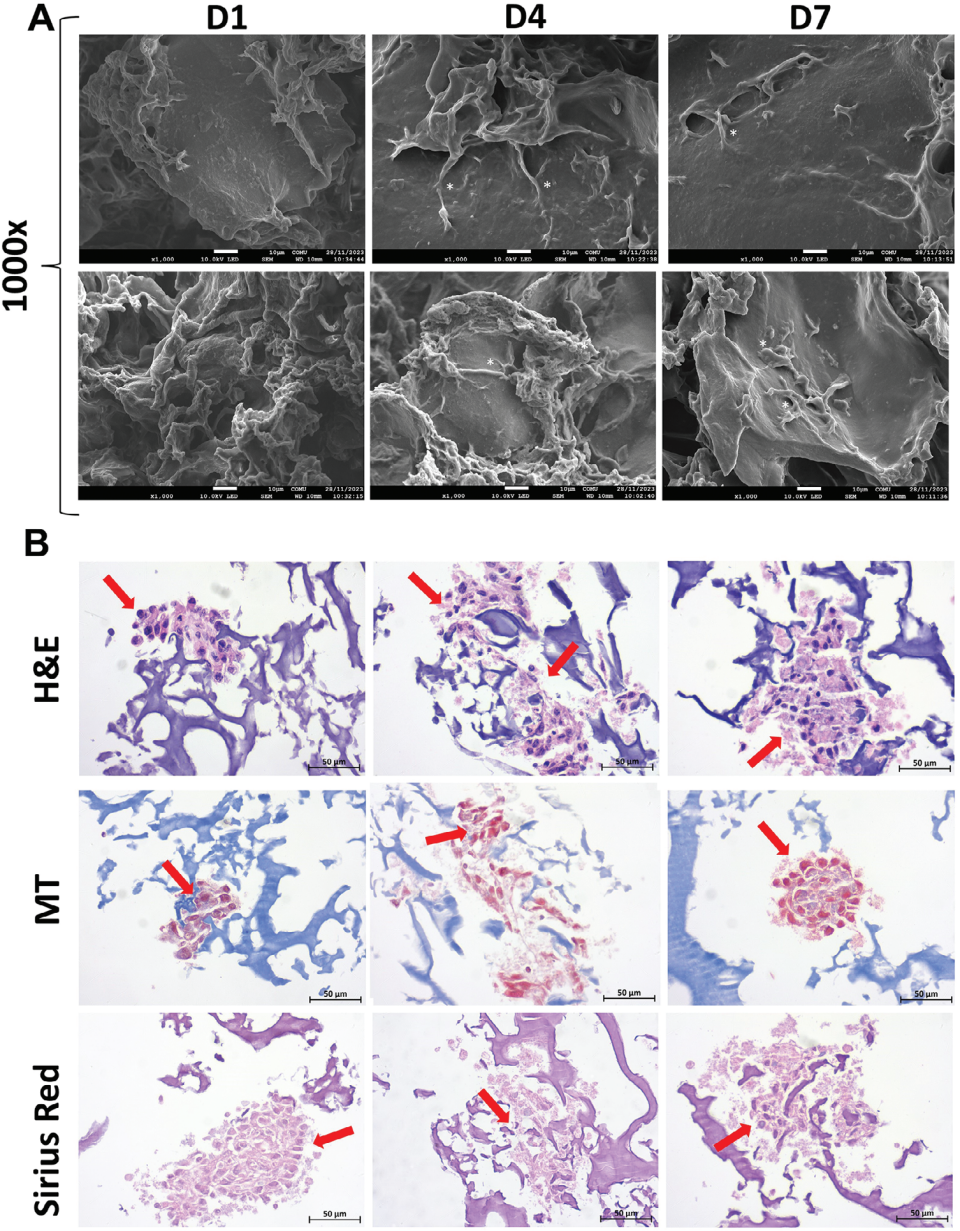
SEM micrographs at x1000 magnifications of C2C12 cells seeded on S4K2 hydrogel‐based scaffolds for 1, 4, and 7 days (white scale bars represent 10 µm) A). Histological images of H&E, MT, and Sirius red staining for in vitro myogenic differentiation of C2C12 cells cultured within S4K2 hydrogel‐based scaffolds for 1, 4, and 7 days. The black scale bar is 50 µm. Red arrows indicate the cells in the matrix B).

**Figure** **6**A shows the SEM micrographs of the cell‐seeded scaffold sections at 1, 4, and 7 days. At each time point of culture, myoblasts seemed to spread throughout the surface of the S4K2 hydrogel‐based scaffolds, indicating that they were suitable hosts for cell growth and proliferation.

In the histochemical analysis, S4K2 hydrogel‐based scaffold cryosections were stained with H&E, MT, and Sirius red to examine cellular structures and collagen formation on the cell‐laden constructs on days 1, 4, and 7 (Figure 6B). H&E staining revealed that the S4K2 hydrogel‐based scaffold provided proper cell infiltration, growth, and development. Moreover, MT and Sirius red staining showed collagen deposition within the S4K2 hydrogel‐based scaffolds. Depending on the extraction method, the resulting SS can have different amino acid compositions, which could affect cell viability and collagen secretion. Sulfur‐containing amino acids, such as methionine and cysteine in hot water‐extracted SS are highly related to promoting the proliferation and attachment of different mammalian cell lines and collagen production.^[^ ^33^, ^58^, ^59^ ^]^ Within this mind, it can be suggested that wool keratin with a high cysteine residue content could exhibit a similar effect. Based on the collagen production in the histological sections between the 1st and 7th days of in vitro culture, we conclude that the S4K2 hydrogel‐based scaffolds can benefit the boost of skeletal muscle, mainly composed of collagen.

### 2.6 Myogenic Differentiation‐Related Gene Expressions

**Figure 7:**
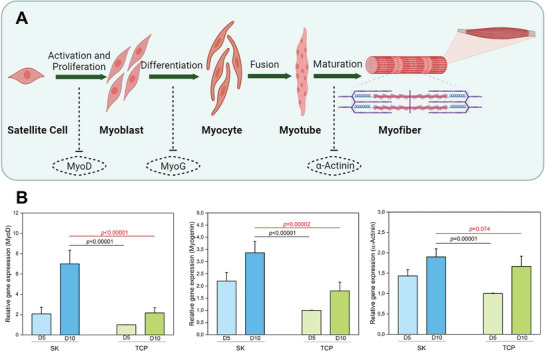
Schematic representation of myogenic differentiation steps A). Quantitative expression levels of myogenic genes, including MyoD, MyoG, and α‐actinin, related to C2C12 cells within the S4K2 hydrogel‐based scaffolds for 5 and 10 days. *p* ≤ 0.05 represents a significant difference within and among the groups B).

Myogenic differentiation is a highly organized sequential process during which muscle precursors called myoblasts differentiate to form mature skeletal muscle through various myogenic regulatory factors, such as MyoD and MyoG^[^ ^60^ ^]^ (**Figure** **7**A). MyoD is highly expressed during the formation of mononuclear myocytes from myoblasts in the early stage of differentiation. At the same time, MyoG plays an active role during the last stage of differentiation and the fusion of myotubes into multinucleated myotubes.^[^ ^61^ ^]^ Eventually, myotubes mature into myofibers by expressing the muscle‐specific protein α‐actinin.^[^ ^62^ ^]^ Based on this phenomenon, RT‐qPCR was performed to assess the expression levels of myogenic differentiation genes, as shown in Table S3 (Supporting Information). Figure 7B demonstrates the relative expression levels of myogenic genes, including MyoD, MyoG, and α‐actinin, at scheduled time intervals (days 5 and 10). As the experimental days progressed, the remarkable upregulation of MyoD and Myogenin (MyoG) genes in S4K2 hydrogel‐based scaffolds compared to TCP. These results imply that our S4K2 hydrogel, with an increased surface area and smaller pore structure, supported cell–cell signaling and subsequent myogenic differentiation.^[^ ^63^ ^]^ In contrast, on the 10th day of culture, there were no statistically significant differences between the levels of the α‐actinin gene expressions associated with myotube transformation in S4K2 and TCP (*p* = 0.074). Considering the anisotropic organization of natural skeletal muscle, the biomaterial intended for skeletal muscle regeneration must induce 3D cellular alignment and elongated myotube formation. Along with optimal pore size and distribution, the anisotropic internal 3D microarchitecture and the highly aligned structure are critical factors in achieving this goal.^[^ ^5^, ^53^ ^]^ The S4K2 hydrogel, with an appropriate pore range, supported myoblast proliferation and differentiation up to a certain level but did not sufficiently enhance myogenic maturation. The insufficient differentiation between myoblasts and myotubes is ascribed to the inadequate alignment and superorganization structure of the S4K2 hydrogel.

Some in vivo studies have explored using natural polymer‐based hydrogels, such as WK^[^ ^15^ ^]^ and SS^[^ ^30^ ^]^ for skeletal muscle regeneration and found that these materials enhanced the myoblasts' adhesion, proliferation, and integration into the host muscle, which is crucial for the formation of new myofibers. Considering these findings, it can be proposed that our porous S4K2 hydrogel‐based scaffold can be a good candidate in muscle tissue engineering applications as it offers a synergistic effect of SS and WK on myoblast proliferation and differentiation.

## 3 Conclusion

This work sought to fabricate novel porous hydrogel‐based scaffolds containing WK and SS using an in situ bubble‐forming hydrogel entrapment process. The EDC/NHS coupling reaction used in this process offers several advantages, such as creating a porous structure by removing entrapped CO_2_ bubbles generated during rapid gelation and producing a strongly crosslinked polymer network. Hydrogels with varying amounts of SS and WK were produced to determine the hydrogel exhibiting optimal structural and mechanical properties for the material design that can be used for skeletal muscle regeneration. After comprehensive evaluations, S4K2 hydrogel‐based scaffolds were determined to be the most suitable materials in terms of porosity, pore size, adequate flexible structure, and durability. Moreover, they exhibited a higher material recovery rate during cyclic loading–unloading experiments, suggesting they possess suitable micromechanical strength to support contractile functionality, which is crucial for mimicking the native skeletal muscle environment. In addition, they showed promising results in neovascularization, myoblast proliferation, attachment, and differentiation. This can be attributed to the favorable pore structure facilitating the exchange of nutrients and oxygen alongside the well‐known biocompatible and cell‐adhesion properties inherent to the WK and SS comprising the hydrogel. Skeletal muscles inherently exhibit a highly hierarchical and anisotropic morphology. Thus, the biomaterial designed for effective regeneration must be capable of addressing this requirement. RT‐qPCR results showed that S4K2 hydrogel with a suitable pore range supported the early and last stages of myogenic differentiation. However, the lack of superorganization and aligned structure prevented myotube formation and maturation, limiting the potential use of this synthesis strategy in muscle tissue engineering applications. Integrating this specific design into our hydrogels is our primary objective, as we want to achieve a better mimicry of natural tissue structure and function in future studies. Many methods for designing aligned structures are proposed in the literature, and among these, we will use the temperature gradient lyophilization technique that can be most suitable for our strategy. Furthermore, after confirming 3D cellular alignment and elongation through fluorescent staining of cell‐laden hydrogel‐based scaffolds, we plan to assess the capability of these scaffolds for functional recovery in in vivo models of VML. In conclusion, our study on material design and myoblast behavior can be considered a preliminary study of future works on implantable scaffolds for skeletal muscle regeneration.

## 4 Experimental Section

### Materials

Bombyx Mori silkworm cocoons were purchased from Koza Han (Bursa, Türkiye). Raw wool samples were provided by local wool suppliers in Çanakkale, Türkiye. *N*‐(3‐dimethylaminopropyl)‐*N*′‐ethylcarbodiimide hydrochloride (EDC, 8.00907.0025), *N*‐hydroxysuccinimide (NHS, 98%), proteinase K from Tritirachium (lyophilized powder, ≥30 units mg^−1^ protein) were purchased Merck (Germany).

### Extraction of Silk Sericin (SS)

**Figure 8:**
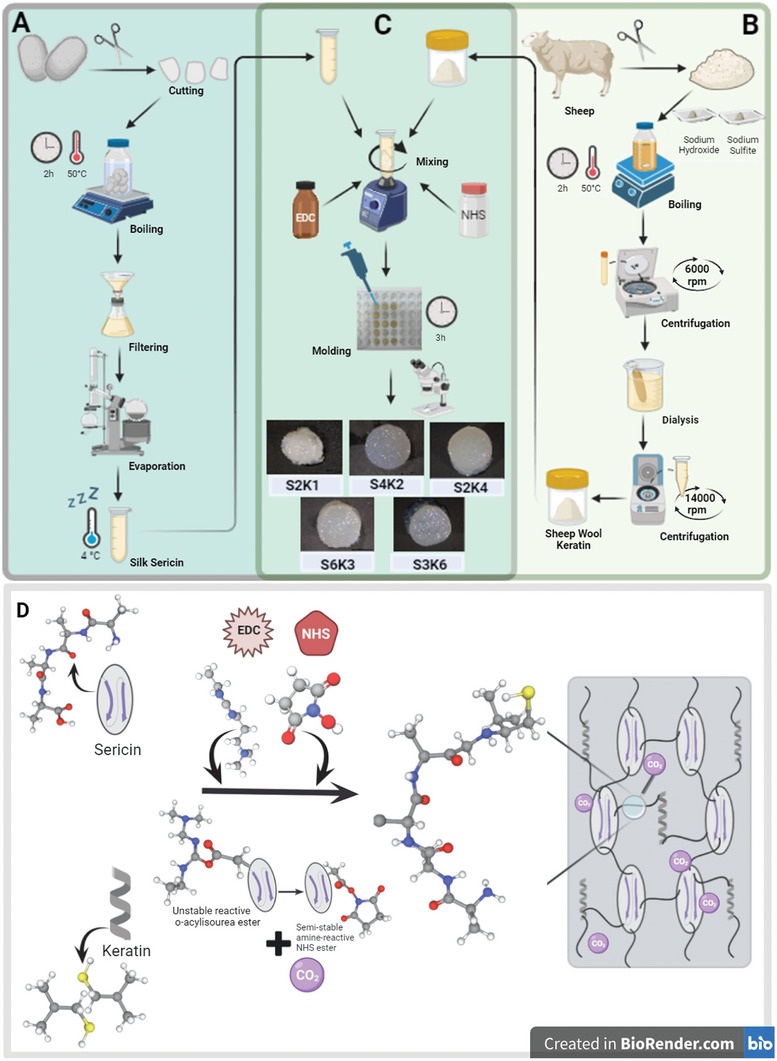
Schematic representation of producing a porous sericin/keratin hydrogel‐based scaffold using an in situ bubble‐forming strategy. The overall process for water‐extracted sericin from Bombyx Mori A). Keratin extraction and purification process from sheep wool B). Preparation of five hydrogel‐based scaffolds containing different ratios of sericin and keratin C). An overview of the mechanism for in situ bubble‐forming strategy. During the EDC/NHS coupling reaction, an amide bond is formed between carboxyl and amine groups in the proteinous mixture; in the meanwhile, CO_2_ and intermediates are released due to the activation of carboxyl groups in the sericin with EDC and NHS D).

SS was extracted from Bombyx mori silkworm by the previously described method with a moderate modification.^[^ ^64^ ^]^ Briefly, silk cocoons (4 g) were chopped into small pieces, then boiled in 1 L of deionized water at 100 °C for 120 min. The resulting mixture was then filtered to remove insoluble particles. The solution was evaporated using a rotary evaporator (Buchi rotavapor R‐210, Switzerland) under a reduced pressure of 43 mbar and at 25 °C until the final concentration was achieved at 6% w/v. Viscous SS stock solution was stored at 4 °C when not used (**Figure** **8**A).

### Extraction and Purification Process of Wool Keratin (WK)

Keratin was extracted from wool samples using a sulfitolysis reaction according to the method described by the group.^[^ ^31^ ^]^ Briefly, defatted and clean wool fibers were cut into small pieces and then subjected to an alkaline extraction solution (100 mL) containing NaOH (5%, w/v) and Na_2_SO_3_ (5%, w/v). The reaction took place at 50 °C for 2 h under dynamic conditions in a bench‐top shaker incubator (Benchmark Scientific, Incu‐Shaker Mini, USA). After the extraction, the resulting mixture underwent purification steps such as filtration, centrifugation, and dialysis. The dialysate was then lyophilized and stored as a powder at ≤−20 °C for further use (Figure 8B).

### Biochemical Analyses of Protein Extracts

Protein content in the WK was determined by Qubit 4 Fluorometer (Thermo Fisher Scientific) using a Qubit Protein BR Assay Kit (Invitrogen, A50668) according to the manufacturer's instructions. Briefly, individual stock solutions at concentrations of 1–20 mg mL^−1^ were prepared by dissolving the keratin extracts in ultrapure water. Each WK solution was mixed with necessary reagents in the kit and incubated for 10 min at room temperature (RT) in a dark place. After the fluorescence readings were completed, a calibration plot was obtained by measuring the relative fluorescence units (RFU) over the concentrations of WK. The protein content of the unknown samples was then calculated from the resulting calibration curve (Figure S1‐A, Supporting Information).

Total free thiol (*─*SH groups) content formed regarding disulfide bond ruptured in the WK extracts was determined spectrophotometrically using Ellman's reagent according to the methods reported previously.^[^ ^31^, ^65^ ^]^ Equation (1) given below, obtained from the calibration curve formed using external standards of L‐cysteine (Figure S1‐B, Supporting Information), was used to calculate the amount of free *─*SH groups present in the WK extract

(1) $$\begin{array}{ccc} & & \mathrm{Free}\;\mathrm{SH}\;\mathrm{Groups}\;\frac{\left(\mathrm{mmol}\right)}{\mathrm{Keratin}\left(\mathrm{mg}\right)}\\ & & \;=\frac{\left[\left(\frac{\mathrm{OD}412}{\mathrm{slope}}\right)x\left(\mathrm{Diution}\;\mathrm{Factor}\;x\;\mathrm{Total}\;\mathrm{Volume}\right)\right]}{\mathrm{Keratin}\;\mathrm{Weight}\;\left(\mathrm{mg}\right)} \end{array}$$

Lowry protein assay was also applied to determine the concentration of the SS using the bovine serum albumin (BSA) standard calibration graph and method given in the previous study.^[^ ^31^ ^]^ Additionally, SDS‐PAGE was performed to determine the MW distribution of the polymer chains in the WK and SS extracts. For this purpose, the Laemmli method was used to prepare 4% stacking and 12.5% resolving gels. Amounts of the keratin, BSA and sericin loaded onto per lane were 25, 0.5, and 125 µg, respectively. The necessary processes for preparing, running, and staining the gel and visualizing protein bands were performed as in the previous studies.^[^ ^31^, ^66^ ^]^

### Preparation of SS/WK Hydrogels‐Based Scaffolds

To obtain porous 3D SS/WK hydrogel‐based scaffolds, the in situ bubble‐forming hydrogel entrapment process via EDC/NHS coupling reaction was applied as in the previously reported method with a moderate modification.^[^ ^12^ ^]^ First, SS solutions at concentrations of 2% w/v, 3% w/v, and 4% w/v were prepared by diluting the SS stock solution (6% w/v) with ultrapure water. Subsequently, WK was dissolved in 1 mL of SS solution (2%, w/v) at a concentration of 1% w/v by vortex mixing. Thus, a solution containing a total of 30 mg protein was obtained. 82.8 mg EDC and 20.16 mg NHS were added to this solution, mixed vigorously, and immediately molded into a custom‐made 48‐well polytetrafluoroethylene (PTFE). With the addition of EDC/NHS, CO_2_ bubbles were generated within seconds, showing that the gelation was initiated. The resulting gel was left to rest at RT for 3 h for maturation. Then, matured hydrogels were washed thoroughly with distilled water, frozen at −40 °C and lyophilized overnight. Finally, a porous SS (2%) and WK (1%) hydrogel‐based scaffold (referred the S2K1) was obtained. Specifically, the S2K1 hydrogel‐based scaffold comprises 2% w/w SS and 1% w/w WK. A spectrum of hydrogel‐based scaffolds containing varying amounts of WK and SS (S4K2, S2K4, S6K3, and S3K6) were also prepared by following the same reaction steps (Figure 8C) to investigate the effects of component ratio on the structural and mechanical characteristics of emerging hydrogels and to determine the optimum component ratio. Moreover, the amounts of EDC/NHS increased proportionally with the total protein content. The reaction mechanism of the in situ bubble‐forming strategy between the SS and WK hydrogel network is also presented in Figure 8D.

### Physicochemical Characterizations of Hydrogel‐Based Scaffolds

Structural analysis of the materials was carried out using attenuated total reflection‐Fourier transform infrared (ATR‐FTIR), where spectra were recorded by scanning 4000–500 cm^−1^ wavenumbers with a 16 cm^−1^ spectral resolution on a Nicolet IS50 Flex Gold Infrared Spectrometer (Thermo Fisher Scientific, USA).

X‐ray diffraction (XRD) studies were conducted to determine the crystallinity degree of materials by exposing them to Cu‐Kα radiation (*λ* = 1.54 056 Å, 45 kV, and 40 mA) with a 2θ angle range of 5°–70° and a scan speed of 0.02° min^−1^.

Thermogravimetric analysis was carried out with a PerkinElmer device (TGA 8000, USA) to determine the mass loss of samples heated between 30 and 800 °C with a heating rate of 10 °C min^−1^ under dry nitrogen flow.

The total surface areas of the hydrogel‐based scaffolds were determined using the multipoint Brunauer–Emmet–Teller (BET) method with a Quadsord SI BET instrument (Quantachrome Instruments, Anton‐Paar GmbH, Austria). Following a degassing process at 90 °C, samples were subjected to a nitrogen gas atmosphere at 77 K within the relative pressure range of 0.01–0.99 P/P0.

The swelling ability of hydrogel‐based scaffolds was evaluated by immersing them in PBS (10 mL, pH 7.4) for 24 h. The swelling ratios (%) of the samples were then calculated using Equation (2) given below

(2) $$\mathrm{Swelling}\;\mathrm{Ratio}\;\left(\%\right)\;\;\left(\frac{\mathrm{mg}}{\mathrm{mg}}\right)=\frac{m_{\mathrm{wet}\;}-\;m_{\mathrm{dry}}}{m_{\mathrm{dry}}}\;\;x\;100$$

where *m*_wet_ is the weight of the swollen hydrogel, and *m*_dry_ is the initial weight of the hydrogel.

Contact angle measurement was performed to assess the wettability properties of the material surface by using Attension Theta Optical Tensiometer (Biolin Scientific, Gothenburg, Sweden). The process was completed by dispensing 5 µL of water from a motorized syringe with ± 1° precision onto the material surface and then recording the water absorption rates.

Rheological measurements were conducted to reveal essential aspects of gel structure and mechanical behavior using a DHR 2 Rheometer (TA Instruments, USA). For this purpose, matured hydrogels were placed between 40 mm diameter cross‐hatched parallel plates, with a 1.0 mm gap. An oscillatory amplitude sweep between 0.01% and 100% was performed at 1 Hz frequency and 25 °C to determine the linear viscoelastic region (LVR) to select the strain value to be used subsequently. Then oscillatory frequency sweep was carried out using 4% within the LVR at a frequency range of 0.1–100 Hz at 25 °C. The temperature sweep between 25 and 40 °C was conducted with a heat rate of 1 °C min^−1^, 4% strain, and at 1 Hz frequency. Finally, gel formation was monitored with an applied time sweep protocol for 30 min at 7.5% strain within the LVR, 1 Hz frequency, and 25 °C after adding each sample in pregel form onto the rheometer plate. Storage modulus (*G*') (Pa) and loss modulus (*G*'') (Pa) were recorded.

Uniaxial compression tests of the wet hydrogels were conducted to assess the mechanical durability using a micromechanical testing device (Univert CellScale Biomaterials Testing, Canada). Hydrogels (cylindrical, *ø* = 4.31 mm; *h* = 7 mm) were placed between two plates and compressed with a 50N load cell at a rate of 0.05 mm s^−1^ until 85%–90% of their initial thickness. To assess the elasticity of the wet hydrogels, a cyclic compression test was also performed with a strain rate of 9 mm min% under 75% stress with 50 loading–unloading cycles. All measurements were carried out at RT, and force‐displacement data were processed to obtain stress–strain curves, revealing material properties, such as Young's modulus and compression strength values. Young's modulus values were calculated using the graphical method as given in Figure S2 (Supporting Information). Additionally, real‐time imaging of the samples was conducted using a tripod‐mounted camera (Logitech, HD 1080p) operating at 5 Hz frequency throughout all experiments.

Field emission scanning electron microscopy (FE‐SEM JFM 7100F EDS, JEOL, Japan) (FE‐SEM) analysis was conducted to determine pore structure, average pore size, and pore size distribution of the hydrogel‐based scaffolds. On the other hand, the quantitative elemental composition of the material's surface was determined by an energy dispersive X‐ray (EDX) analysis. Before imaging of cell‐loaded scaffolds, samples were fixed in 2.5% v/v glutaraldehyde prepared in PBS (pH 7.2–7.4) for at least 24 h, washed with distilled water, dehydrated through a graded series of ethanol (50%–100%), and then air‐dried. All the specimens were sputter‐coated with Gold/Palladium (Au:Pd 80:20%) for 90s to obtain high quality and resolution images with different magnification levels at 10 kV in a high vacuum. Average pore size and distribution were also calculated from 20 to 40 pores in a sample randomly selected by processing the SEM images using ImageJ software (Version 6.0.0.260, NIH).

### In Vitro Biodegradation Study of Hydrogel‐Based Scaffolds

In vitro biodegradability tests of hydrogel‐based scaffolds were carried out by following the method described in the previous studies.^[^ ^31^, ^34^ ^]^ Briefly, lyophilized hydrogel‐based scaffolds (*n* = 3) were accurately weighed (m*i*) and then incubated in proteinase K solution (0.01%, w/v) prepared in Tris‐HCl buffer (0.02 m, pH 8.0) at 37 °C for different time points up to 10 days by changing the medium every 2 days. The samples taken out at each time point (3, 7, and 10 days) were rinsed with distilled water, freeze‐dried, and reweighted (m*f*). The degradation rate (%) was calculated using Equation (3) given below

(3) $$\mathrm{Weight}\;\mathrm{Loss}\;\left(\%\right)=\left(\frac{m_{i}-m_{f}}{m_{i}}\right)\;x\;100$$

### In Ovo Chick Chorioallantoic Membrane (CAM) Assay

In ovo CAM assay was used as an in vivo alternative animal model to investigate proangiogenic properties and biocompatibility of the hydrogel‐based scaffolds as previously described in the studies with minor modifications.^[^ ^34^, ^67^, ^68^ ^]^ Fertilized chicken eggs purchased from a local farm (Çanakkale, Türkiye) were transported to the laboratory in temperature‐controlled conditions and cleaned with 20% ethanol. The eggs were incubated vertically in a forced‐draft constant‐humidity incubator (Brinsea Ovation 28 Advance Ex, UK) at 37.5 °C and 60% relative humidity (RH) by rotating every 120 min for 3 days. On day three of embryonic development (EDD3), a window was opened from the sharp end of the egg, and this opening was sealed with cleaned parafilm to avoid dehydration and microbial contamination of the egg content. Windowed eggs were reincubated for another 7 days under the same conditions by checking their viability daily. At the end of the EDD10, sterilized samples were implanted between in central allantoic veins on the CAM surface and imaged using a stereo microscope (Stemi 305, Zeiss) with a digital camera (Axiocam 105 color, Zeiss). To assess the angiogenic effects of samples, filter discs saturated with 10 µL vascular endothelial growth factor (10 ng µL^−1^, VEGF) and PBS were also used as positive and negative controls, respectively. At the end of the testing period (EDD14), images of CAM‐scaffold complexes were processed using Image J software to obtain vascular density between the foveal and parafoveal regions located within the 1 and 2 mm imaginary area surrounding the grafts, respectively. Then, the blood vessel indexes were calculated as the ratio of vascular density at EDD14 to EDD10. After imaging, CAM‐biomaterial complexes were excised and prepared for hematoxylin and eosin staining (H&E) and SEM analysis by routine protocols.^[^ ^68^ ^]^ Additionally, chick embryos that have not reached the 15th day of their development would not experience pain and also are not considered alive before the 17th day.^[^ ^69^, ^70^ ^]^ Ethical approval is unnecessary as all experiments were completed within 14 days.

### Cell Culture Studies—Cells and Culture Conditions

The hydrogel‐based scaffolds were exposed to UV light (254 nm) for 60 min for sterilization before cell seeding. C2C12 mouse myoblast cells purchased from the American Type Culture Collection (CRL‐1772TM, ATCC, VA) were cultured in Dulbecco's modified Eagle's medium (DMEM, GibcoBRL, Gaithersburg, MD) supplemented with 5% fetal bovine serum (FBS, GibcoBRL), 1% L‐glutamine (1%), and antibiotic–antimycotic solution (1%) under standard culture conditions (5% CO_2_, 95% humidity, and 37 °C). Myoblast cells at passages 5–7 were seeded onto the scaffold surface in varying cell densities for different experimental setups, such as viability and proliferation tests (100 000 cells per scaffold), histological assessments (250 000 cells per scaffold), and gene expression studies (250 000 cells per scaffold). Cell‐loaded scaffolds were incubated in the same culture conditions for 1, 3, and 7 days by replacing the medium every 2–3 days with an equal volume of fresh medium. Additionally, tissue culture polystyrene (TCP) was used as the control group for each assay.

### Cell Culture Studies—Cell Viability and Proliferation Assay

To evaluate the biocompatibility of the hydrogel‐based scaffolds regarding cell viability and proliferation at the indicated time points, live/dead and MTS assays were applied as in a previous study.^[^ ^71^ ^]^ For the live/dead assay (Molecular Probes, Thermo Fisher, UK), cell‐seeded scaffolds were washed with PBS, after which they were incubated with 4 µm calcein‐AM (green fluorescein marker) and 2 µm ethidium homodimer‐1(EthD‐1, red fluorescein marker) for 10 min in the dark at 37 °C and 5% CO_2_. Subsequently, live cells (stained green) and dead cells (stained red) were observed using a fluorescence microscope (Leica DMIL, Germany) with excitation wavelengths of 488 and 527 nm, respectively.

Moreover, cell proliferation on hydrogel‐based scaffolds was quantitatively measured by using an MTS assay. For this purpose, cell‐seeded scaffolds were washed with PBS and incubated with MTS reagent for 1 h at 37 °C. Absorbance measurements related to the number of viable cells in the scaffolds were conducted using a spectrophotometer (Thermo Scientific, Multiskan Sky Microplate Spectrophotometer) at 490 nm.

### Cell Culture Studies—Histochemical Analyses

Cellular structures and responses toward the hydrogel‐based scaffolds were investigated by histological analysis. Initially, specimens were washed with PBS and fixed overnight in a 10% neutral buffered formalin solution, dehydrated through ethanol series, and embedded in paraffin. Sections (3–5 µm) were prepared on poly‐l‐lysine microscope slides (Thermo Scientific, Braunschweig, Germany) and stained with H&E for histomorphological assessment. Besides, collagen deposition within scaffolds was qualitatively analyzed by Masson trichrome (MT) and Sirius red staining using routine staining protocols described in previous studies.^[^ ^54^, ^72^ ^]^ Images of sections were taken via a camera‐attached light microscope (Zeiss Primostar integrated with Axiocam 105 color camera, Jena, Germany).

### Cell Culture Studies—Quantitative Real‐Time Polymerase Chain Reaction (RT‐qPCR) Analyses on Myogenic Differentiation

How hydrogel‐based scaffolds affect muscle tissue formation by employing real time‐quantitative polymerase chain reaction (RT‐qPCR) is investigated. Specifically, the expression levels of musculogenic markers Myogenin, α‐Actinin, and MyoD are analyzed. Following a 5‐and 10‐days of culture of C2C12 cells on scaffolds, RNA was extracted using a GeneDireX kit (USA) and converted into cDNA using a Bio‐Rad kit (USA). RT‐qPCR was conducted using a Bio‐Rad CFX96 thermal cycler (USA). The primer sequences obtained (Oligomer Biotechnology Jsc., Turkey) for gene amplification are detailed in Table S3 (Supporting Information).

### Statistical Analysis

The results were presented as means ± standard deviation and processed using Microsoft 365 Apps for the enterprise. Quantitative assays were performed in triplicate for all experiments. Differences among the groups were evaluated using OriginPro 2024 (v10.1.0.170, Learning Edition, Origin Lab Corporation, MA) software by two‐way analysis of variance (ANOVA) followed by Tukey's test. The *p* ≤ 0.05 was considered to show a statistically significant difference.

## Conflict of Interest

In the study, the proposed method for keratin extraction from wool is related to a patent application (TPE 2014/02104, Turkish Patent & Trademark Office), which corresponds to Dr. Yavuz Emre Arslan (Ph.D.) and Dr. Tugba Sezgin Arslan (Ph.D.).

## Supporting information



Supporting Information

Supplemental Video 1

Supplemental Video 2

## Data Availability

The data that support the findings of this study are available from the corresponding author upon reasonable request.
