# Supplementary material for: A Facile Strategy for Preparing Flexible and Porous Hydrogel‐Based Scaffolds from Silk Sericin/Wool Keratin by In Situ Bubble‐Forming for Muscle Tissue Engineering Applications
Source: Macromol Biosci. 2024 Oct 20;25(2):2400362. doi: 10.1002/mabi.202400362 (PMC11827552; doi:10.1002/mabi.202400362)
Supplement: Supplementary file 1 — Supporting Information [file MABI-25-2400362-s003.docx]

**Supporting Information**

**A Facile Strategy for Preparing Flexible and Porous Hydrogel-based Scaffolds from Silk Sericin/Wool Keratin by In-Situ Bubble-Forming for Muscle Tissue Engineering Applications**

Elif Beyza Demiray^1^, Tugba Sezgin Arslan^1^, Burak Derkus^2^, and Yavuz Emre Arslan^1^*

^1^Regenerative Biomaterials Laboratory, Department of Bioengineering, Faculty of Engineering, Çanakkale Onsekiz Mart University, Çanakkale 17100, Turkey

^2^Stem Cell Research Lab, Department of Chemistry, Faculty of Science, Ankara University, Ankara 06100, Turkey

*Corresponding author:

E-mail: [emre.arslan@comu.edu.tr](mailto:emre.arslan@comu.edu.tr) (Y. E. Arslan)

Tel.: +90-286-218-0018; Fax: +90-286-218-0541


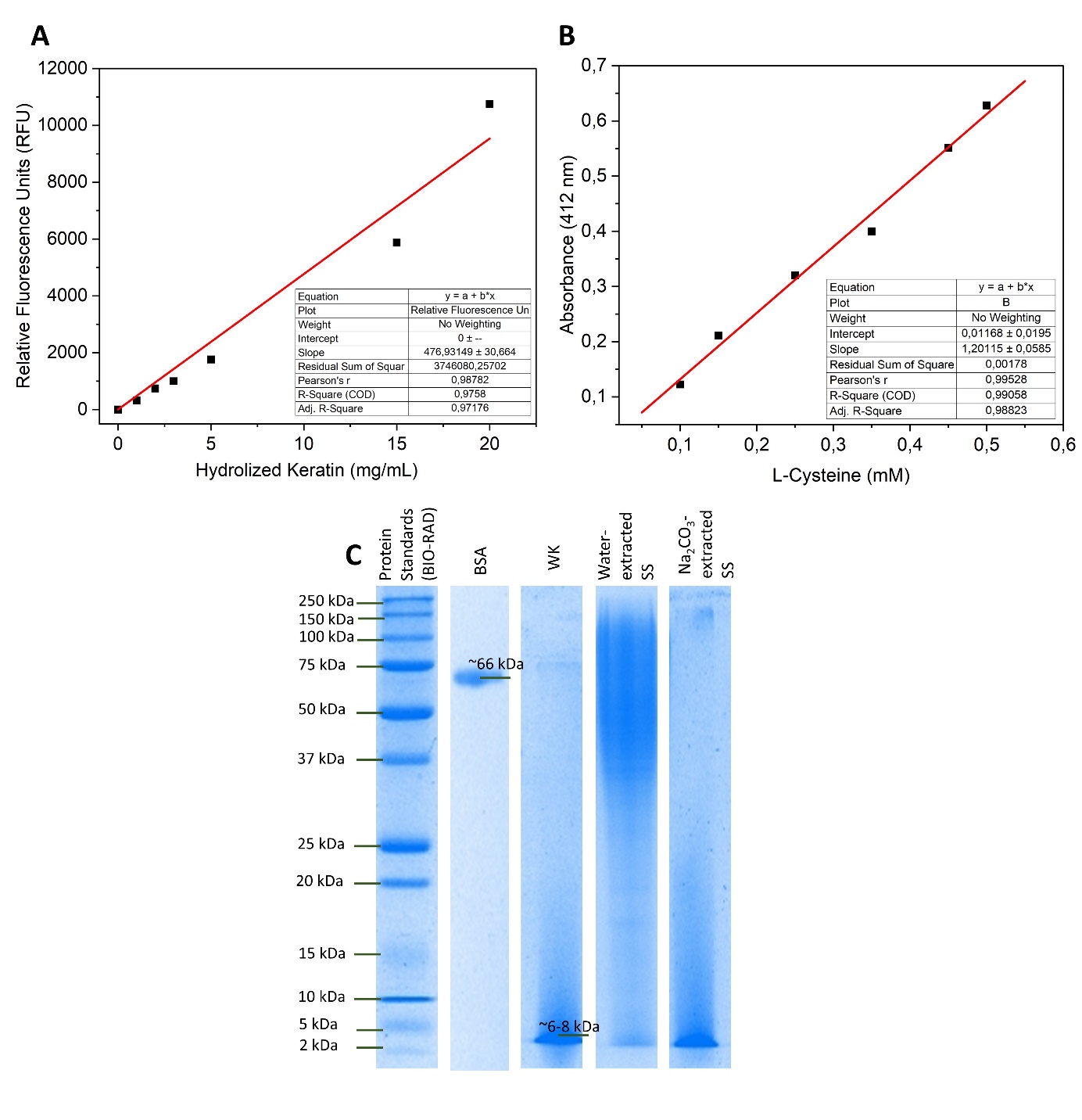


**Figure S1.** The standard calibration curves for Qubit protein (A), Ellman's assay (B), and the SDS-PAGE pattern of keratin, water-extracted sericin, and Na_2_CO_3_-extracted sericin (C).


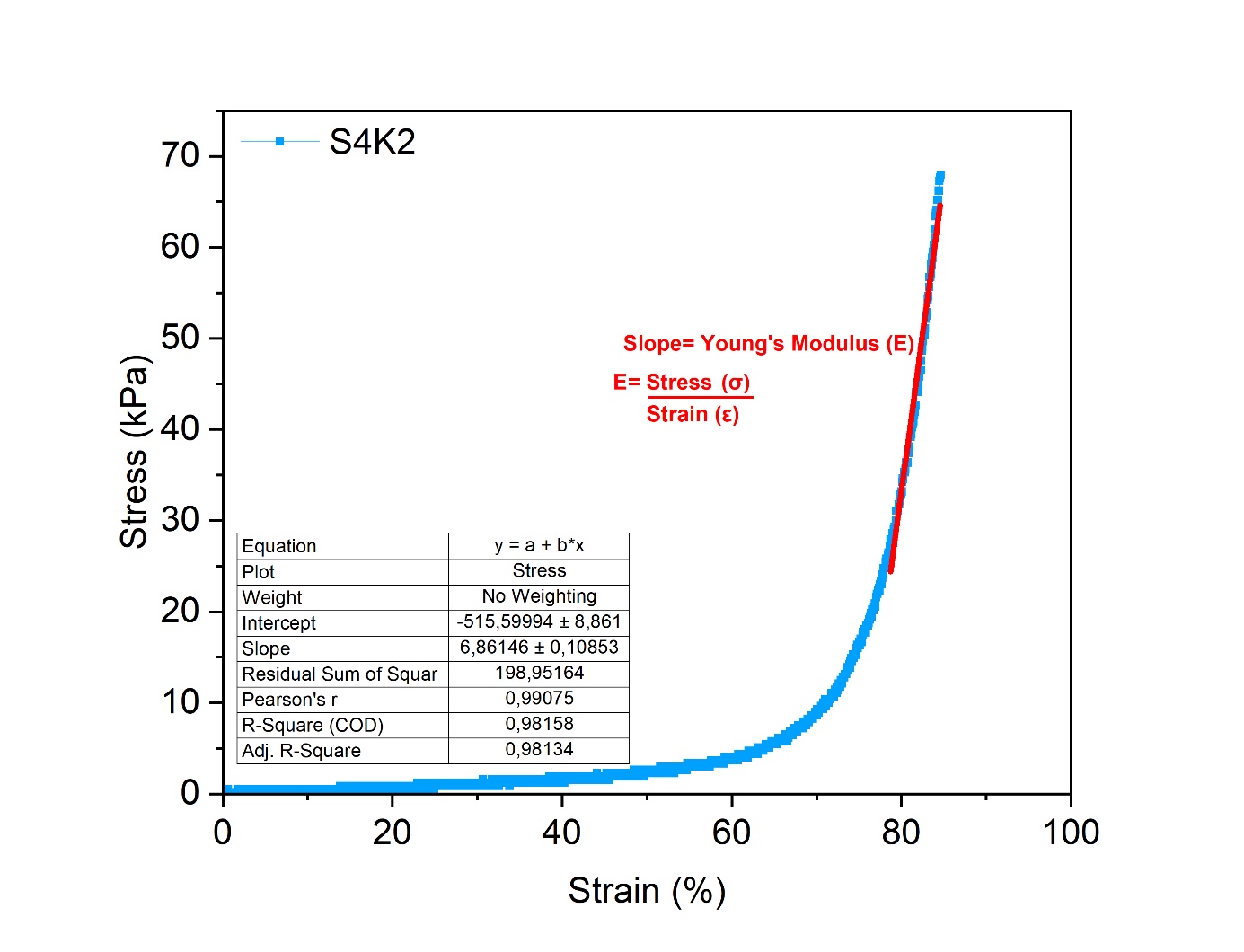


**Figure S2.** Determination of Young’s modulus with graphical method.


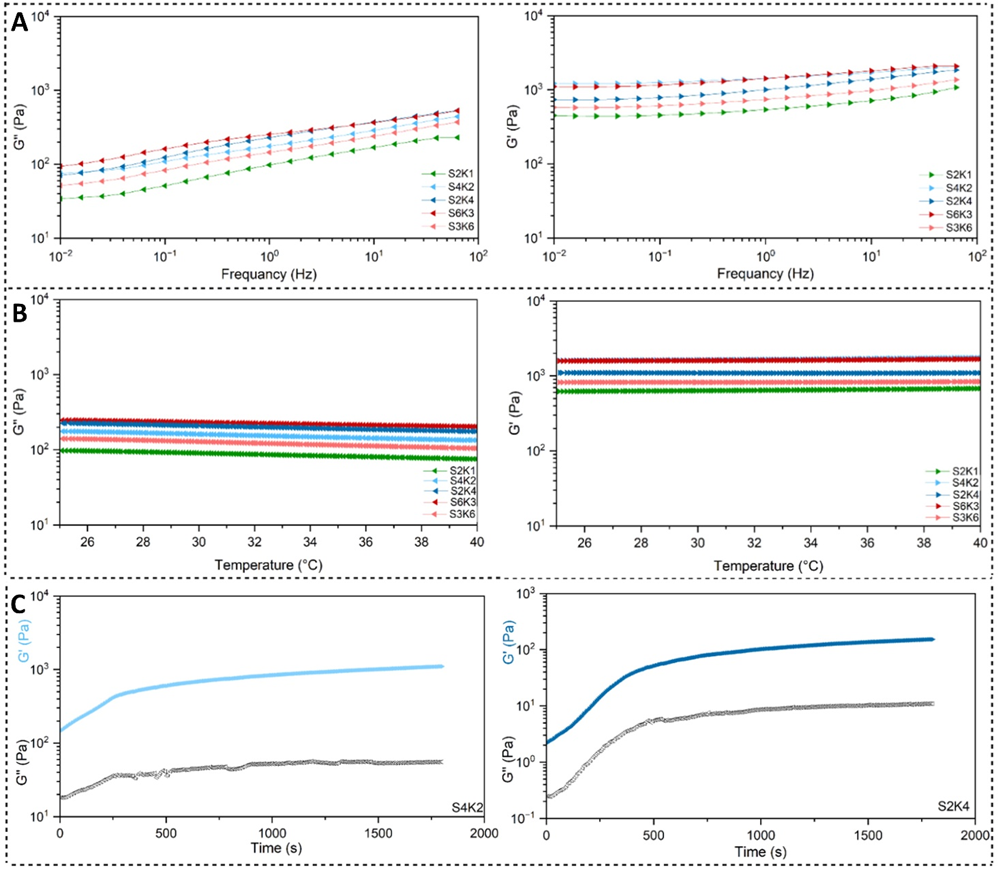


**Figure S3.** Rheological behavior of five different sericin/keratin hydrogels. Graphs demonstrate dynamic modulus (G’ and G’’) values from frequency (A), temperature (B), and time (C) sweeps.


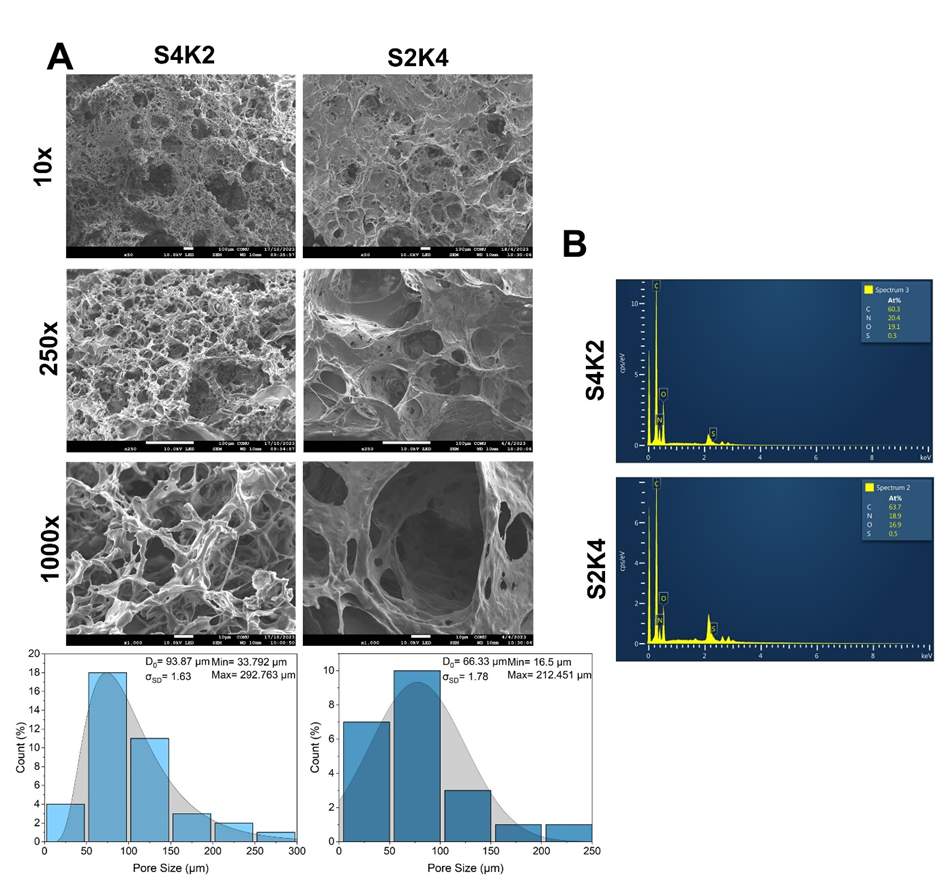


**Figure S4.** SEM micrographs and pore size distribution histograms (white scale bars represent 100 µm for 10x and 250x, and 100 µm for 1000x) (A), EDX spectra (B) of S2K4 and S4K2 hydrogel-based scaffolds.


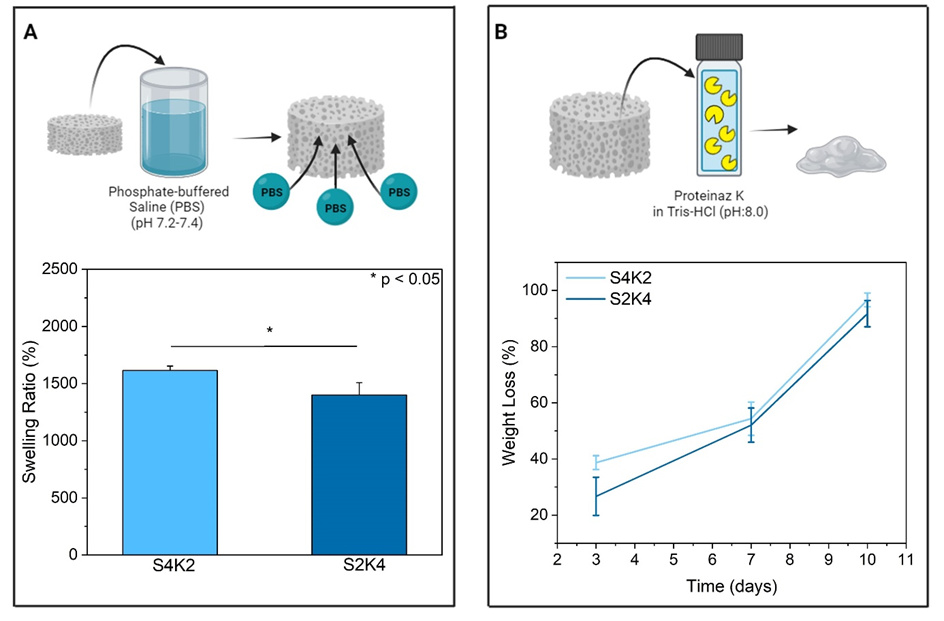


**Figure S5.** Graphs demonstrating swelling ratios (A) and in vitro biodegradation rate (B) of S2K4 and S4K2 hydrogel-based scaffolds.

**Table S1.** Strain, stress, and Young’s modulus values of the samples.

| **SAMPLE** | **STRAIN (%)** | **STRESS (kPa)** | **YOUNG’S MODULUS (kPa)** |
| --- | --- | --- | --- |
| S2K1 | 81.81 ±1.37 | 19.95 ±1.52 | 134.43 ±19.03 |
| S4K2 | 83.33 ±1.43 | 70.68 ±3.83 | 644.14 ±42 |
| S2K4 | 87.5 ±0.57 | 57.8 ±4.61 | 445.26 ±49.18 |
| S6K3 | 88.89 ± 1.3 | 88.34 ±9.46 | 802.48 ±21.95 |
| S3K6 | 83.33 ±1.19 | 55.59 ±3.39 | 471.9 ±43.25 |

**Table S2.** Results of BET analysis of S2K4 and S4K2 hydrogel-based scaffolds.

| **Sample** | **Total Surface Area, BET (m^2^/g)** |
| --- | --- |
| S4K2 | 43.61 |
| S2K4 | 6.66 |

**Table S3.** Primer designs for qRT-PCR analysis

| **TARGET GENE** | **OLIGONUCLEOTIDE SEQUENCES** | |
| --- | --- | --- |
|  | **Forward 5'/3'** | **Reverse 5'/3'** |
| MyoD | 5-GGACTACACTGCCTTCTC-3 | 5-CAGCCTATACTTCAGCCTTTA-3 |
| Myogenin | 5-GGATATGTCTGTTGCCTTC-3 | 5-TGGGTGTTAGCCTTATGT-3 |
| *α*-Actinin | 5-GGACTACACTGCCTTCTC-3 | 5-CAGCCTATACTTCAGCCTTTA-3 |
| GAPDH | 5-TGTTCCAGTATGACTCCACT-3 | 5-TGGTGAAGACACCAGTAGAC-3 |

**Video S1.** Demonstrating rapid recovery of the S4K2 hydrogel under the compression-release process.

**Video S2.** Demonstrating wettability performance of S4K2 hydrogel by using contact angle measurements.
